# Aesthetic Restorations: The Role of The Dental Hygienist in Professional Maintenance: In Vitro Microbiological Study

**DOI:** 10.3390/ma16041373

**Published:** 2023-02-06

**Authors:** Lorenza Francesca Di Petto, Giulia Tetè, Mara Hera, Elisabetta Polizzi

**Affiliations:** 1 Center for Oral Hygiene and Prevention, IRCCS San Raffaele Hospital, Vita-Salute University, 20132 Milan, Italy; 2 Department of Dentistry, IRCCS San Raffaele Hospital, Dental School, Vita-Salute San Raffaele University, 20132 Milan, Italy; 3 Dental School, Vita Salute University, 20132 Milan, Italy; 4 Department of Dentistry, Dental School, IRCCS San Raffaele Hospital, Vita-Salute San Raffaele University, 20132 Milan, Italy

**Keywords:** aesthetic restorations, dental hygiene, cup, Air-Flow, Clearfil Twist DIA

## Abstract

The aim of this in vitro study is to try to identify a treatment in the field of professional oral hygiene techniques that is ideal and less invasive with regard to prosthetic restorations, chosen in lithium disilicate and feldspar ceramics. Seventeen veneers were prepared (eight in lithium disilicate and nine in feldspar ceramic) and each of them was attached to an extracted tooth. The treatment groups were initially contaminated in the Microbiology Laboratory of Università Vita—Salute San Raffaele. After 24 h they were treated with four different methods of professional oral hygiene and were then sent to the Microscopy Laboratory, in which they were analysed by SEM, with 180X, 250X, and 500X magnification, to assess any damage caused to the treated surfaces. The SEM analysis produced 64 images that were evaluated by an experienced dentist, and the use of the prophetic cup seemed to be the most suitable among the evaluated treatments. However, from a microbiological point of view, this method was the least effective in terms of reducing bacterial growth. In this regard, the use of a prophylactic rubber cup combined with a 0.20% chlorhexidine gel is recommended during professional oral hygiene sessions for the maintenance of aesthetic prosthetic restorations.

## 1. Introduction

In the age of communication, smiles play a major role in interpersonal relationships.

Never like in this historical moment have aesthetics assumed such a fundamental importance. Indeed, a recent study points out that, due to the lockdown for the COVID-19 pandemic, cosmetic surgery operations have increased by 15%. The desire to restart after months spent at home has also increased the need for external renewal; moreover, communication through digital platforms has also led to a greater attention paid by the population to their external appearance [1,2].

Having a beautiful smile plays a key role in the perception that we have of ourselves; moreover, the pleasant appearance given by a beautiful smile facilitates and breaks down any barrier between us and others. In addition, a harmonious smile can help you feel more comfortable in everyday life because it increases self-esteem, both in the professional sphere and in any other relational activity [3,4,5,6,7,8,9,10].

The dental aesthetic takes care of the aesthetics of the smile, forming an integral and substantial part of the facial aesthetics. Indeed, it is a branch of dentistry that deals with modifying or removing the imperfections of the smile, which, as mentioned above, is considered a real business card of a person [11,12,13,14,15].

There are many techniques of intervention, from the most basic, such as the removal of extrinsic stains on dental surfaces, to the most complex and advanced dental whitening techniques. Some aesthetic techniques are implemented with adhesive modes and therefore there is no need to file the teeth; others, instead, are more invasive, such as the removal of old amalgam fillings and subsequent replacement with materials with excellent aesthetic performance, or the modification of the shape of the teeth with crowns or veneers made with different aesthetic materials [16,17,18].

The medium- and long-term success of aesthetic restorations is guaranteed by three main factors: patient compliance, personalised and specific follow-up, and the correct choice of oral hygiene techniques for professional maintenance.

Indeed, patients with aesthetic prosthetic rehabilitations is included in long-term follow-up programmes, whose protocol includes manoeuvres of quarterly and/or bi-annual professional oral hygiene, with precise instructions on the most suitable techniques and aids for domestic oral hygiene [19,20,21].

Therefore, the work of the dental team aims to acquire the necessary scientific knowledge about the properties and characteristics of the various devices to be used in a professional environment, so that they can choose the most suitable devices for each individual case.

The purpose of this work is to compare different methods of professional oral hygiene applied on prosthetic aesthetic elements: dental veneers.

After applying a known bacterial load, we evaluate which is the most effective professional therapy for oral hygiene in terms of removing bacterial biofilm and is the least invasive towards the surface of the prosthetic material.

After the experimental phase, in accordance with the scientific literature, precise and effective indications are provided regarding the most effective and least invasive professional oral hygiene treatment in the long-term maintenance of dental veneers.

## 2. Materials and Methods

### 2.1. Dental Veneers

Starting from the extracted dental elements, four hemi-arches were recreated. Subsequently, the elements on which to pack the aesthetic veneers in lithium disilicate and feldspar material were prosthetically prepared.

**Figure 1:**
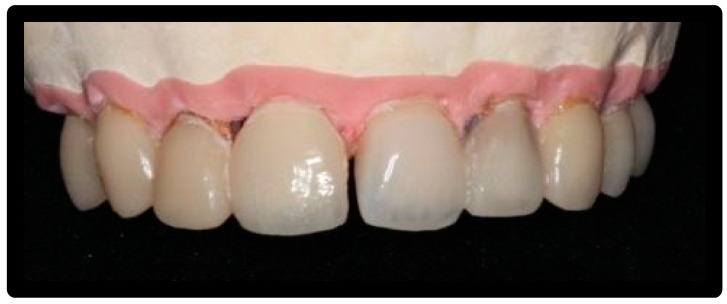
Model 0.

**Figure 2:**
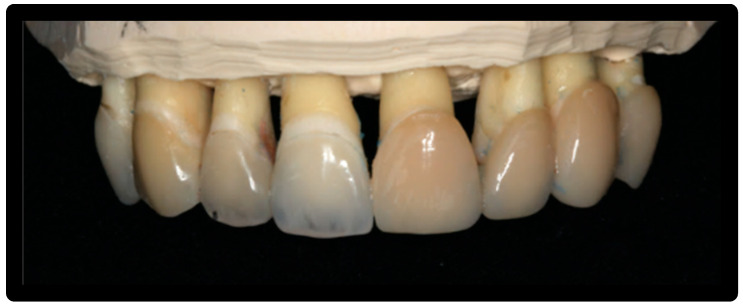
Model X.

Each element was prosthetically prepared using a Chamfer procedure: n° 7 adjacent dental elements for model 0 and n° 8 adjacent dental elements for model X (model 0: 1.4,1.3,1.2,1.1,2.1,2.2,2.3 model X: 1.4,1.3,1.2,1.1,2.1,2.2,2.3,2.4) (Figure 1 and Figure 2).

**Figure 3:**
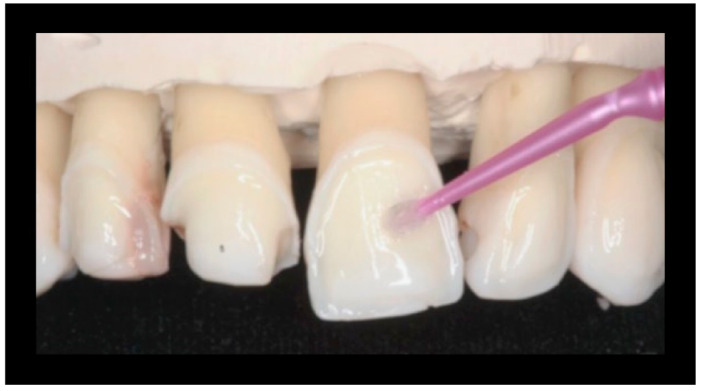
Application of the adhesive.

A total of 15 aesthetic dental veneers were produced (Antonio Lazetera Dental Laboratory), 7 in lithium disilicate material and 8 in feldspathic material, each of which was fixed to the corresponding element extracted with Relix automix cement—Unicem 2, as per protocol. Clinical prosthesis shown in Figure 3.

### 2.2. Microbiology

A bacterial strain, ATCC 25922 *Escherichia coli*, was used for this study.

The organism was cultured on 5% Columbia blood agar plates (bd bbl stacker plates) and incubated at 35 °C overnight.

**Figure 4:**
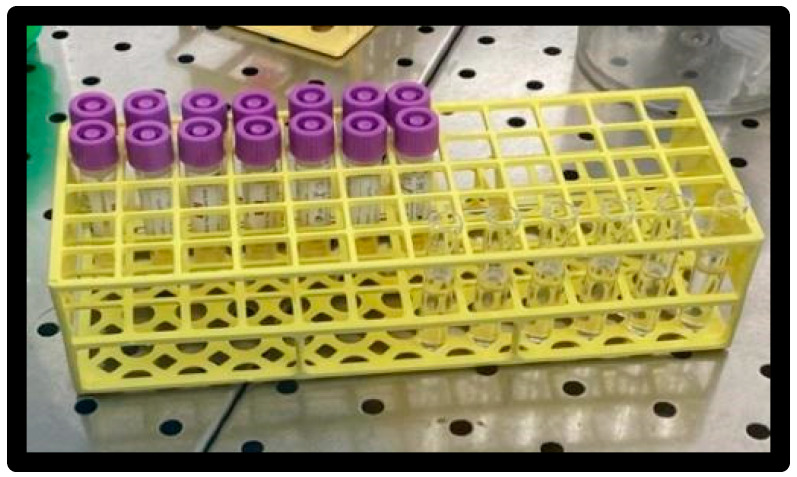
Inoculum vials.

A 0.5 McFarland inoculum of ATCC 25922 was prepared in 3 mL of saline (Figure 4).

### 2.3. Consumables

**Figure 5:**
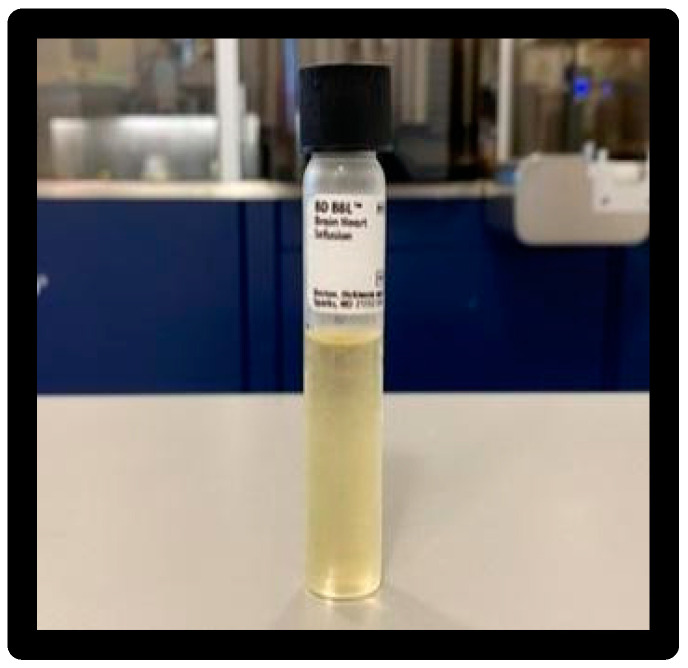
Culture medium.

**Figure 6:**
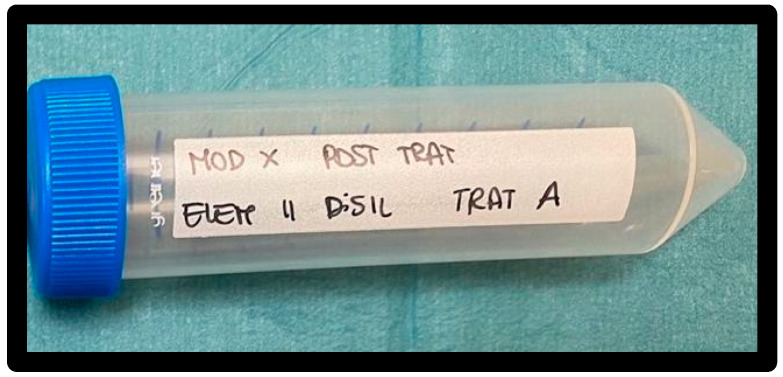
Falcon 50mL.

Sterile cotton swabs, sterile tips (Costar), pipettes, 50 mL tubes (Falcon), and brain heart infusion (BHI) enrichment medium (Figure 5 and Figure 6) were used.

### 2.4. Instrumentation Products

**Figure 7:**
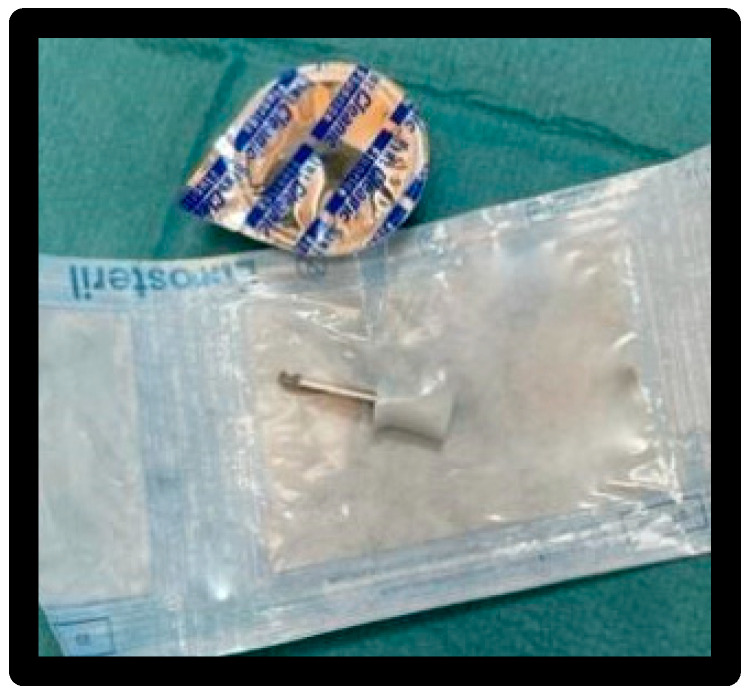
Cup with Cleanic^®^ prophy paste.

- White prophy cup
- Cleanic^®^ prophy paste based on perlite (Figure 7)

- Air-Flow Master Piezon^®^ EMS:

1–5 bar water supply, 5.5–7.5 bar compressed air supply, ultrasonic output max 12 Watts, frequency range 24–32 kHz.

**Figure 8:**
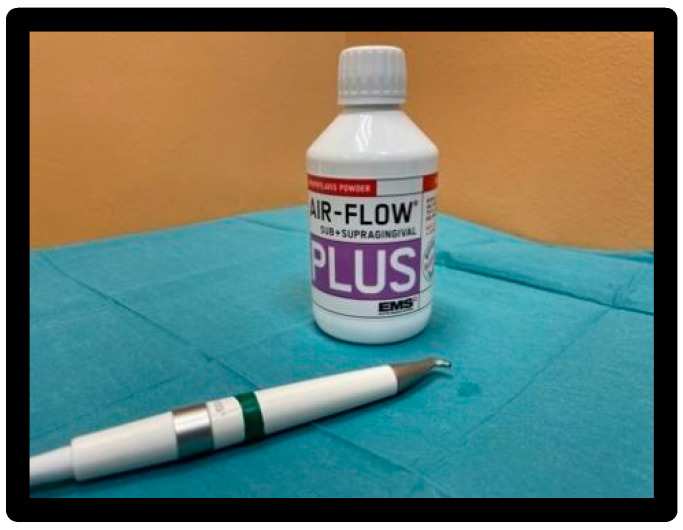
Air-Flow Master Piezon^®^ EMS with Air-Flow Plus^®^ EMS powder.

**Figure 9:**
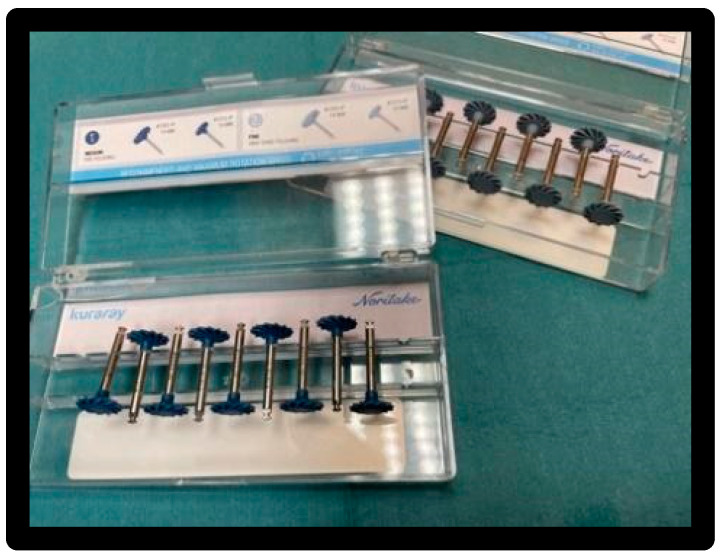
Clearfil Twist DIA rubber pads.

- EMS Air-Flow Plus^®^ powder, based on erythritol, with Chlorhexidine (0.3%) average grain size 14 µm (Figure 8).

- Clearfil Twist DIA polishers in two grain sizes, dark blue and light blue (Figure 9).

### 2.5. Types of Treatments

- Treatment 1: prophy cup + Cleanic paste, 15′/surface at 1200 rpm.
- Treatment 2: Air-Flow with Plus powder (erythritol with CHX) with tip angle of 30 °—60 °, 5 sec/surface at 5 mm distance from the surface.
- Treatment 3: Clearfil Twist DIA rubber pads.
- 1 pass DARK BLUE 15 sec/surface with 10,000 rpm speed.
- 2 pass LIGHT BLUE 30 sec/surface with 10,000 rpm speed.
- Treatment 4: Clearfil Twist DIA rubber pads + irrigation.
- 1 pass DARK BLUE 15 sec/surface with 10,000 rpm speed.
- 2 pass LIGHT BLUE 30 sec/surface with 10,000 rpm speed.

## 3. Methods

### 3.1. Preliminary Phase

A strain of *Escherichia coli* ATCC 25922 was inoculated on a blood agar plate at the Microbiology Laboratory of the Vita Salute San Raffaele University.

After 24 h, a single colony was taken and used to produce the mother culture: CM, pre-inoculated in 3 ml of physiological solution. The CM was prepared to obtain a known concentration of 0.5 McFarland.

### 3.2. Operational Phase

**Figure 10:**
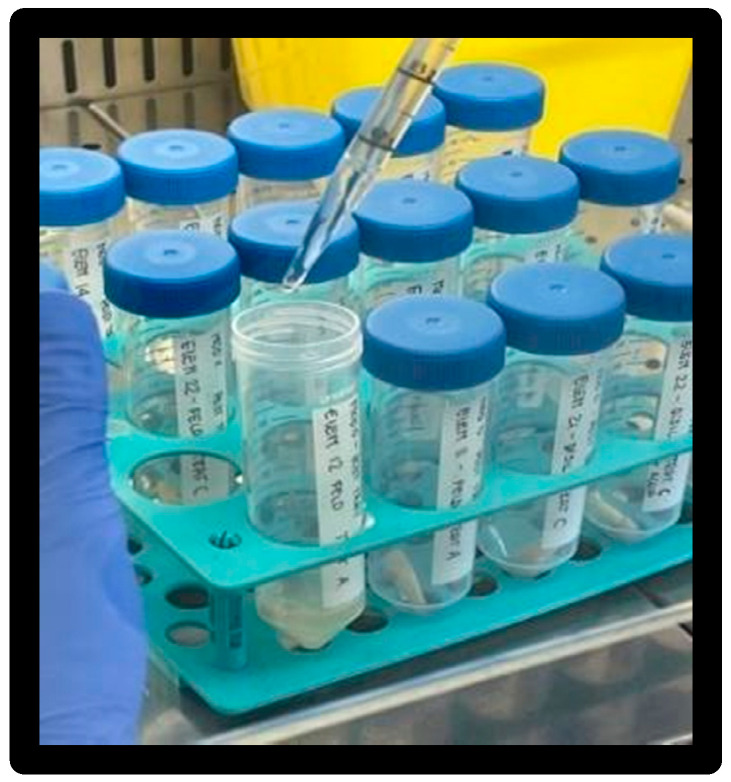
Inoculation of the stock culture.

The dental veneers were contaminated by inserting them into a Falcon and adding the prepared inoculum to each surface (Figure 10).

**Figure 11:**
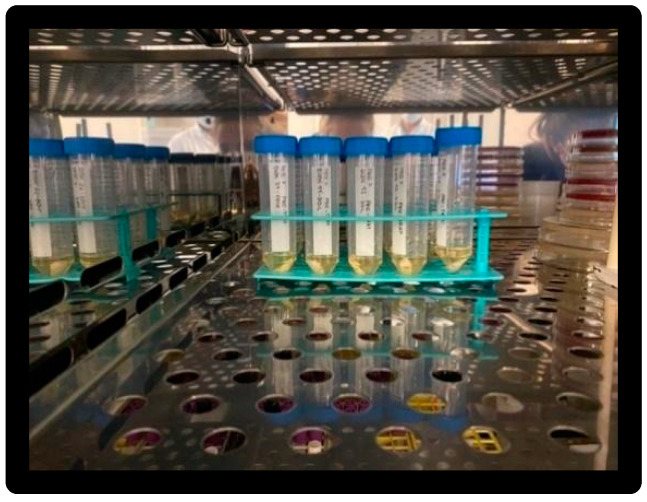
Falcon samples in incubator.

Once the inoculation of all the samples was completed, they were placed in an incubator at 35 ± 2 °C for 24 h (Figure 11).

After 24 h from contamination, the samples were subjected to treatment with the various selected professional oral hygiene methods.

### 3.3. Working Groups

The 15 veneers cemented to the related dental elements through Relix—Unicem 2 automix cement were divided into 4 working groups composed of two veneers in lithium disilicate and two in feldspar. The control group consists of one lithium disilicate and two feldspar veneers.

- GROUP A: Treatment 1—Cup with Cleanic prophy paste.
- GROUP B Treatment 2—Air-Flow with Plus powder.
- GROUP C: Treatment 3—Clearfil Twist DIA rubber pads.
- GROUP D: Treatment 4—Clearfil Twist DIA rubber pads + irrigation.
- GROUP E: Positive control.

### 3.4. Professional Instrumentation Techniques

All professional oral hygiene manoeuvres were performed according to the indications of the literature and the manufacturers of the products and tools used. They were performed by a single operator under standard conditions with a silicone base aid for the stability of the prosthetic elements.

### 3.5. Treatment 1

**Figure 12:**
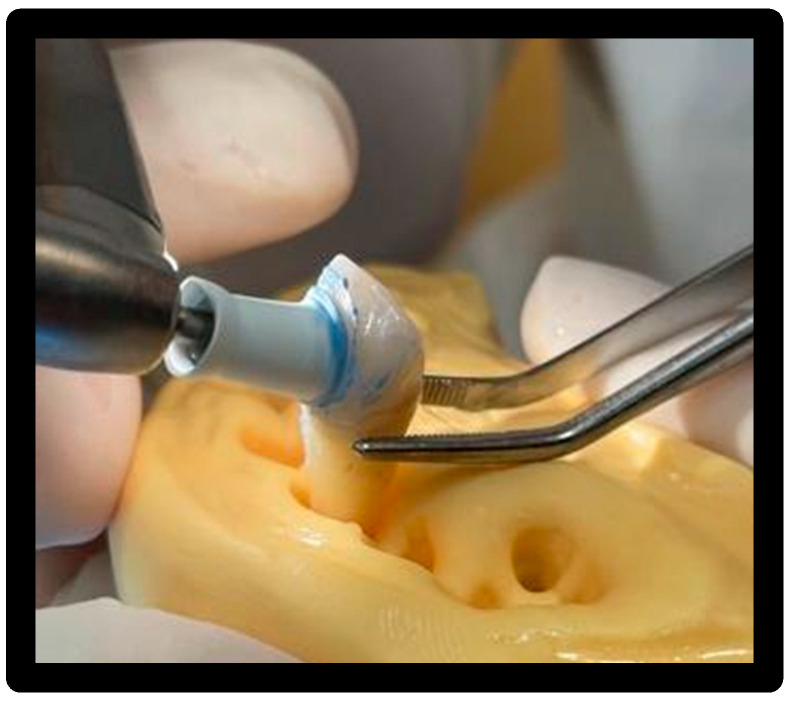
Passing the cup with Cleanic^®^ polishing paste.

- White prophy cup (WIEDER) with Cleanic^®^ prophy paste based on perlite:

Used for 15′/surface at 1200 rpm with circular movements capable of wrapping the entire surface of the veneer (Figure 12).

### 3.6. Treatment 2

**Figure 13:**
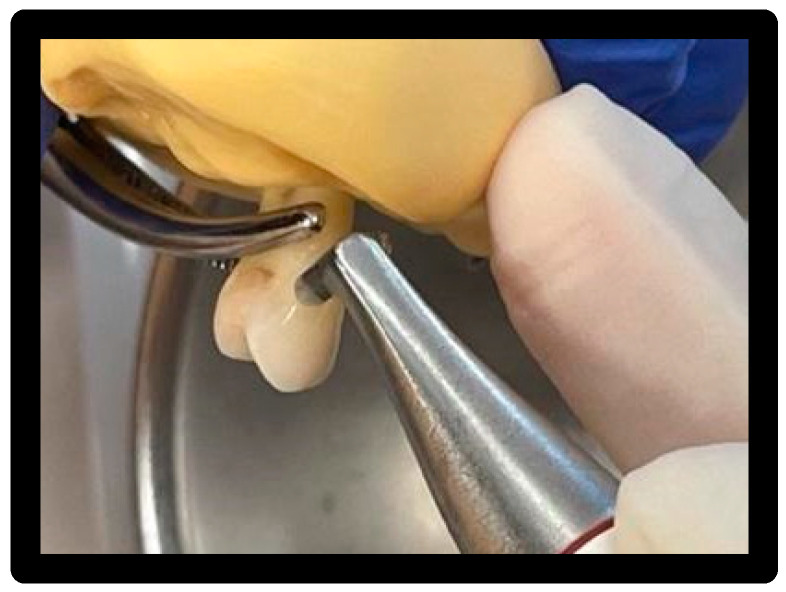
Passage of Air-Flow Master Piezon^®^ EMS using Plus powder.

- Air-Flow Master Piezon^®^ EMS:

1–5 bar water supply, 5.5–7.5 bar compressed air supply, ultrasonic output max 12 Watt, frequency range 24–32 kHz. EMS Air-Flow Plus^®^ powder based on erythritol with CHX (0.3%) grain size 14 μm. Used with the tip of the handpiece, with respect to the dental veneer, at an angle between 30° and 60°; the flow was directed for 5 sec/surface, maintaining a distance of 5 mm from the surface and making a circular movement (Figure 13).

### 3.7. Treatment 3

**Figure 14:**
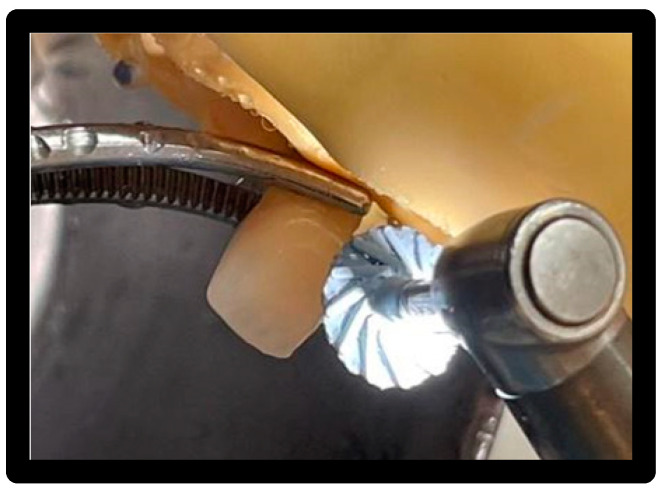
Passage of the dark rubber.

**Figure 15:**
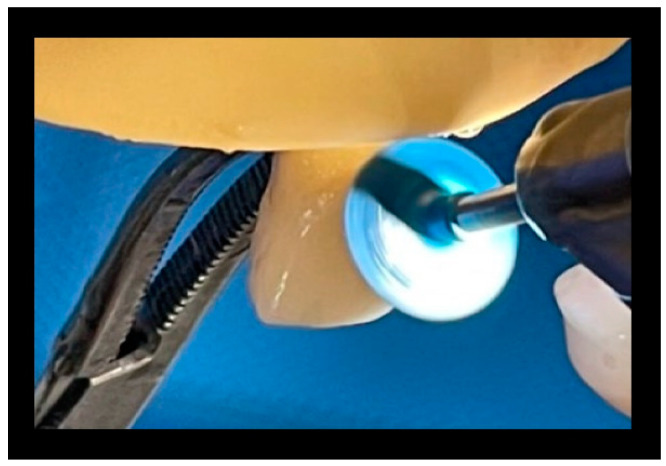
Passage of the light rubber.

- Clearfil Twist DIA—dark blue and light blue:

First pass: dark blue for 15 s on every surface with a speed of 10,000 rpm; second pass: light blue for 30 s on every surface with a speed of 10,000 rpm (Figure 14 and Figure 15).

### 3.8. Treatment 4

**Figure 16:**
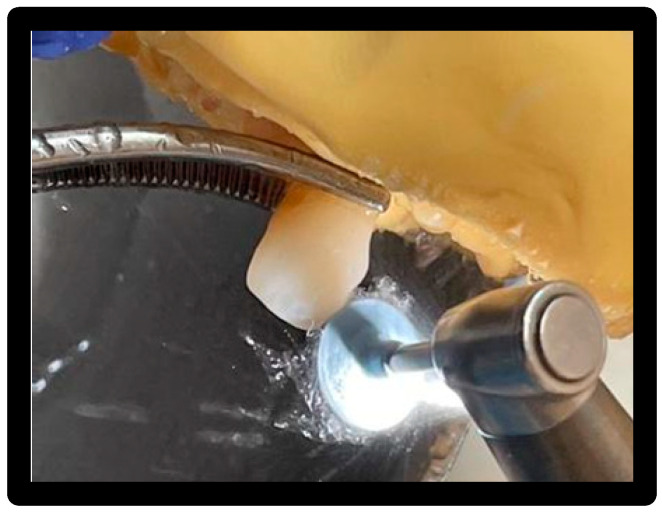
Passage of the dark rubber.

**Figure 17:**
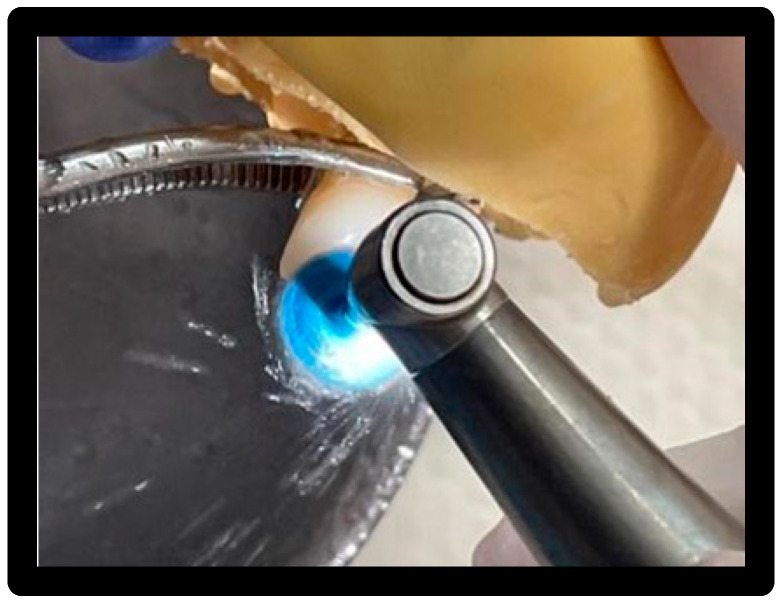
Passage of the light rubber.

- Clearfil Twist DIA—dark blue and light blue with irrigation:

First pass: dark blue for 15 s on each surface with a speed of 10,000 rpm; second pass: light blue for 30 s on each surface with a speed of 10,000 rpm (Figure 16 and Figure 17).

### 3.9. Microbiological Phase

At the end of the treatments, to carry out the evaluations, the veneers were inserted into a new 50 mL Falcon, cultured with the addition of a liquid enrichment medium BHI (brain heart infusion).

**Figure 18:**
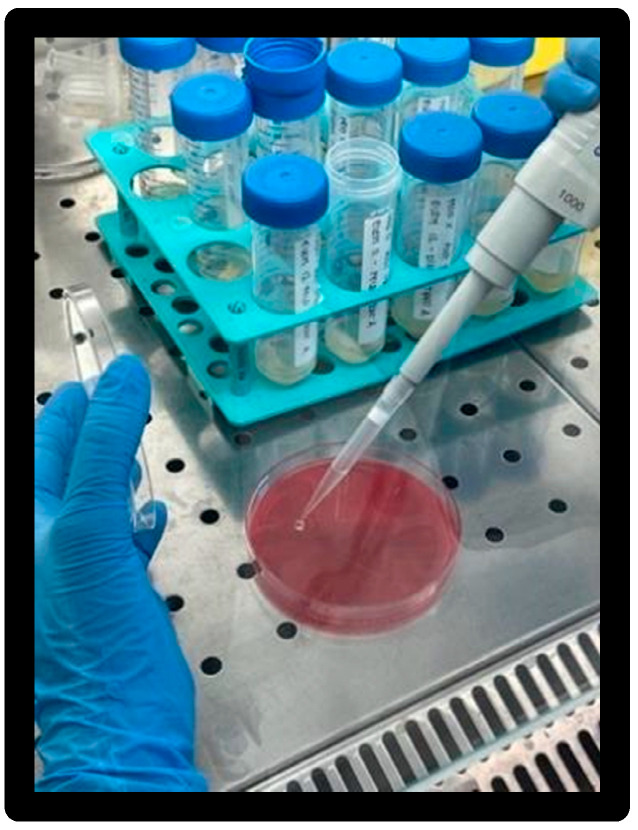
Taking and placing sample on blood agar medium.

After vortexing, the T0 was collected with a duplicate volume of 50 μL and transferred to a blood agar plate and incubated at 35° ± 2 °C (Figure 18).

**Figure 19:**
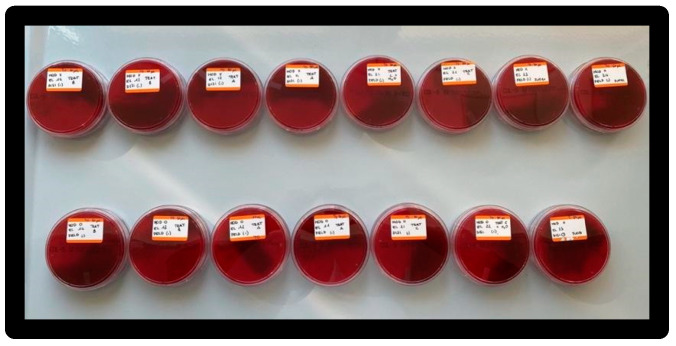
Blood agar plates after incubation; elements of model X at the top, elements of model 0 at the bottom.

After 4 h, to evaluate T1, the operation of taking a sample in duplicate of 50 μL was repeated, and the sample was transferred to a blood agar plate and placed again in incubation at 35° ± 2 °C (Figure 19).

### 3.10. Microscopic Analysis with SEM (Scanning Electron Microscope)

The samples were inserted into the sample order, which was subsequently placed in the machine.

At this point, the sample image was loaded, an area of the sample was focused, and then the entire image was mapped.

Each sample was given a nomenclature called Label.

The analysis was carried out at 5 kV image quality, as the surface was the part being analysed.

On each sample insertion, the correct parameters such as contrast and zoom were manually set.

The lighter points correspond to the heavier elements; vice versa, the darker points represent lighter elements.

Images were taken for each sample at 180×, 250×, and 500×.

**Figure 20:**
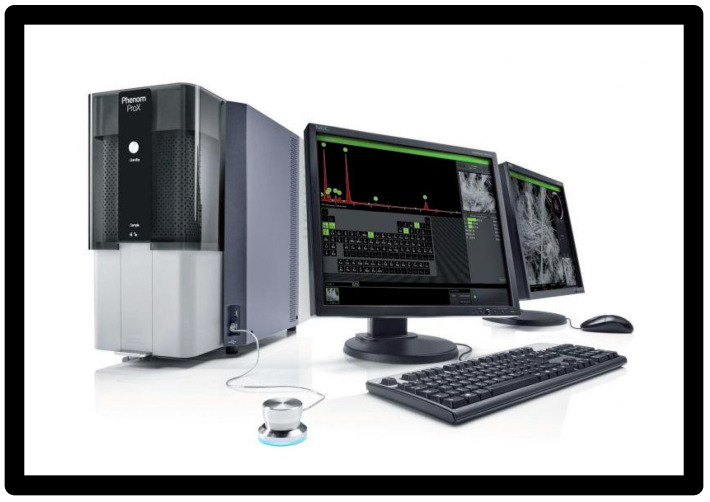
SEM microscope: Phenom Pro G6.

A total of 64 images were shot (Figure 20).

## 4. Results

### 4.1. Microbiological Results

Experiments conducted to assess residual bacterial load after professional oral hygiene treatments showed a lowering in bacterial count on all elements.

Further analysing the obtained data, we evaluated that in the first treatment (cup with prophetic paste), the bacterial load was similar in terms of quantity on cosmetic veneers in feldspar, compared to the same treatment on aesthetic veneers in disilicate at T1. An interesting fact was observed 4 h later at T2, because bacterial growth was greater on the feldspar surface. In addition, for the second treatment (Air-Flow with Plus powder), it can be noted that the results of the reduction in the bacterial load obtained a similar value for rehabilitation in feldspar and disilicate. As for the previous treatment, even in this case it was possible to evaluate a higher growth after 4 h at T2 on aesthetic dental veneers in feldspar.

With regard to the third treatment (Clearfil Twist rubber), a lower charge was obtained on the disilicate prostheses compared to feldspar, but at T2, it was possible to evaluate a different result where the feldspathic veneer showed less bacterial growth.

In the fourth treatment (Clearfil Twist + H2O rubber), an opposite effect was observed, as in the examination of the initial bacterial count, we saw a better result on the feldspar material than the disilicate. In this case, the effect was maintained even at 4 h at T2.

We can therefore evaluate how, in disilicate veneers, the treatment that obtained a better result at T1 was treatment 3 (Clearfil Twist DIA rubbers), while for feldspar, bacterial decontamination was the same in all treatments.

At T2, we can say that the treatment of choice for disilicate is treatment 2 (Air-Flow with Plus powder), while for feldspar, treatment 2 (Air-Flow with Plus powder) and treatment 4 (Clearfil Twist DIA + grommets) have the same effect in maintaining the bacterial load (Table 1 to Table 2).

### 4.2. Micromorphostructural Results

SEM analysis produced 64 images that were evaluated by an experienced dentist.

Each individual element was analysed in its vestibular portion through the SEM microscope to highlight the structural damage resulting from the four professional oral hygiene treatments carried out.

**Figure 21:**
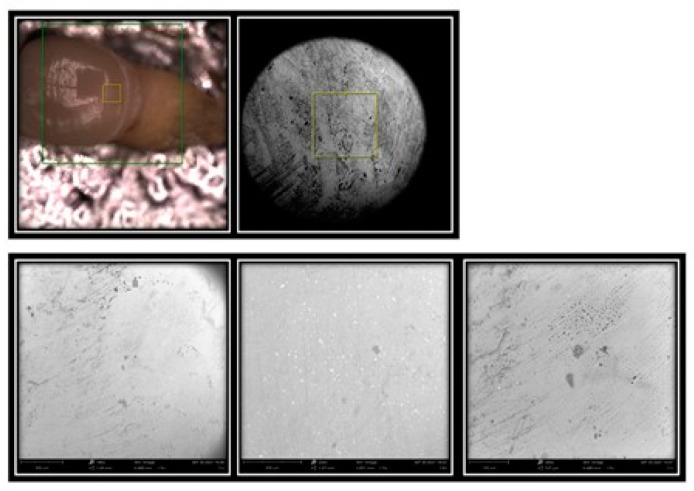
Element 13 of model 0 in disilicate analysed under an SEM microscope at 180×, 250×, and 500× magnifications.

**Figure 22:**
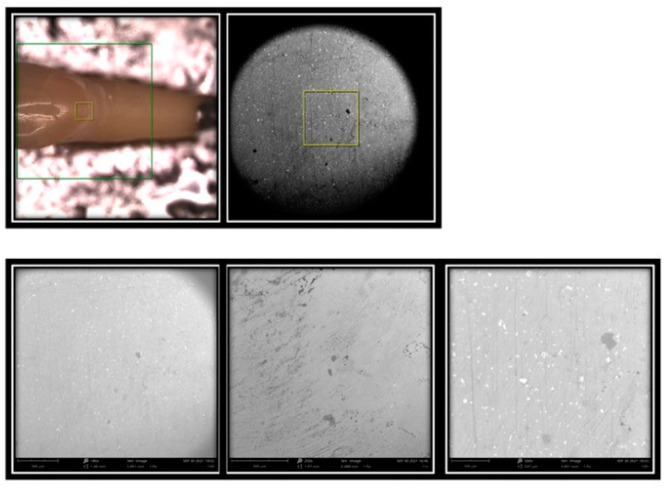
Element 11 of model X in disilicate analysed under an SEM microscope at 180×, 250×, and 500× magnifications.

From the images collected for the disilicate samples treated with treatment 1 (prophylaxis cup with Cleanic paste), it emerged that in the lower magnification (180×), the surface appeared smoother than the surface of the control element. A similar image could be seen at a magnification of 250×, while at 500×, the circular marks left by the passage of the prophetic cup were evident (Figure 21 and Figure 22).

In addition, darker thickening areas were visible, which correspond to micro-dust residues present in the Cleanic paste.

From the images collected for disilicate samples treated with treatment 2 (Air-Flow with Plus powder), it was already clear from the lower 180× magnification that the surface had been polished, while at higher magnifications (250× and 500×), the surface appeared more irregular (with surface roughness) due to the pressure exerted by the air jet.

From the images collected for disilicate samples treated with treatment 3 and 4 (Clearfil Twist DIA without water for treatment 3 and Clearfil Twist DIA with water for treatment 4), the surface looked very smooth, especially in treatment 4, due to the abrasion of the first layer of material. Therefore, these treatments are considered more aggressive than others.

From the images collected for feldspathic ceramic samples treated with treatment 1 (prophylaxis cup with Cleanic paste), it emerged from the lower magnification (180×) that the surface was smooth and completely polished. At maximum magnification (500×), surface irregularities due to micro-dust residues in the Cleanic paste were noted. In addition, it emerged that the texture of the ceramic veneer was completely preserved.

Images of feldspar samples treated with treatment 2 (Air-Flow with Plus powder) showed darker areas due to the approach of the Air-Flow jet and lighter areas due to dust residues on the surface.

Feldspar samples treated with treatment 3 and 4 (Clearfil Twist DIA without water for treatment 3 and Clearfil Twist DIA with water for treatment 4) were, from the images obtained by SEM, polished to a level that affected the consistency of the ceramic feldspar.

Therefore, the results obtained under the SEM microscope show that the treatment with prophylaxis cup and Cleanic paste was the least aggressive in both materials of dental veneers, while the treatment with Air-Flow and Plus powder appears more invasive because it creates a less homogeneous surface in both materials used.

Finally, treatments with Clearfil Twist DIA, both with and without water, are the most aggressive of both the examined materials, to a greater extent in the feldspar ceramic veneers.

## 5. Discussion

Today, having a pleasant and healthy appearance is essential for the growth of interpersonal relationships, which are essential in both social and professional life.

The literature mainly describes protocols for proper professional oral hygiene in patients with fixed prosthetic products, but there is little evidence regarding cosmetic restorations [22].

An optimal adhesive restoration has been achieved in particular if the preparation has been fully inserted into the enamel, proper adhesive treatment procedures have been carried out, and a suitable fixing composite has been selected [23].

Professional oral hygiene techniques included in a personalised follow-up and recall program with proper plaque control at home become a guarantee of medium- and long-term success [24,25,26,27].

Tools used in professional oral hygiene, especially when used by inexperienced operators, can cause iatrogenic damage to prosthetic structures [28,29,30,31].

The literature describes another method to reduce gum inflammation and to comply with prosthetic manufacturers, as a laser, and Angiero et al. reported in 2019 you can use a low-level laser therapy neck with a 645 nm diode laser to reduce tissue inflammation and improve the healing phase of tissues.

To ensure the long-term permanence of prosthetic restorations, the dental hygienist must propose the most suitable and least invasive maintenance protocol for the patient.

Our goal, in fact, given the limited scientific evidence, was to verify the least harmful methods for the prosthetic ceramic surfaces with lithium disilicate and feldspar. Comparing them with the microbiological results, we had to verify the method which was the most effective in removing the bacterial biofilm and the least invasive towards the material used.

All the treatments used in the study have led to a reduction in bacterial load, a figure known in the literature with the exception of Clear Fil Twist DIA which, being a new product, has no scientific evidence to certify its effectiveness. The microbiological results showed that the treatment T1 3 (Clear Fil Twist DIA) in lithium disilicate products results in better results in terms of reduction in bacterial load. Since there are no references in the literature, this is not a confirmed result, but it can be an important basis for future research.

While at T1 in feldspar artifacts, treatments were equivalent, it is interesting to note that at T2 after 4 h, there was a reduction in bacterial growth in both feldspar products and lithium disilicate products.

These results clash with the microscopic SEM, but that have logic in the fact that a very invasive tool for the prosthetic surface is also able to efficiently remove bacterial biofilm.

Our results show that lithium disilicate veneers, due to their reduced thickness for aesthetic reasons, are more fragile than feldspar veneers [32].

As stated in the literature, ceramic veneers performed significantly better than indirect laminated composite veneers after a decade, both in terms of survival rate and quality of surviving restorations [33].

From the collected images, in fact, the surface irregularities were more visible in the disilicated samples, especially in the treatments that involved the use of Air-Flow and mechanical instruments with Clearfil Twist water grommets. The literature on the subject agrees in that Air-Flo is mainly used as the gold standard for the decontamination of more resistant surfaces, such as titanium implant surfaces or metal prostheses [34].

On the other hand, the prophylactic cup is the least aggressive towards both materials with which aesthetic prosthetic products have been made; in fact, various traditional polishing systems on materials such as zirconia and feldspar yield excellent results with low risk of wear or surface roughness [35].

Despite the limitations of this study, hoping for an enlargement of the examined sample, one might think that an elective treatment should be less aggressive and at the same time effective in terms of bacterial biofilm removal.

The use of the prophylactic cup, among the treatments evaluated, seems to be the most suitable method. However, as can be seen from microbiological analysis, it does not achieve statistically significant results in all samples in terms of reduced bacterial growth, especially at T2.

In this regard, it is recommended, in professional oral hygiene sessions for the maintenance of aesthetic prosthetic restorations, that hygienists use a rubber cup combined with a 0.20% chlorhexidine gel for prophylaxis.

## 6. Conclusions

Further in vivo and in vitro studies are necessary to verify the effectiveness of the treatment. However, the importance of our study lies precisely in the fact that there is very little evidence in the literature on the maintenance of aesthetic dental veneers and it was very rare to find references that used in vitro study, so this work can provide a basis to support practitioners in their clinical practice and, of course, to expand their knowledge on the subject.

## Figures and Tables

**Table 1:** Table of disilicate veneers results indicated in cfu/mL.

Tooth Element	T0	T1	T2
X 11 disilicate- PROC.1CUP	1.5 × 10^8^	4 × 10^5^	8 × 10^5^
X 12 disilicate- PROC.1CUP	1.5 × 10^8^	1.6 × 10^5^	3.2 × 10^5^
X13 disilicate- PROC. 2 AIR FLOW	1.5 × 10^8^	2.4 × 10^5^	7.2 × 10^5^
X14 disilicate- PROC. 2 AIR FLOW	1.5 × 10^8^	9 × 10^4^	2.7 × 10^5^
021 disilicate- PROC.3 gum	1.5 × 10^8^	1.54 × 10^5^	6.16 × 10^5^
022 disilicate- PROC.3 gum + H2o	1.5 × 10^8^	2.1 × 10^5^	8.4 × 10^5^
023 disilicate- ctr.	1.5 × 10^8^	3.5 × 10^5^	1.4 × 10^4^

**Table 2:** Table of disilicate veneers results indicated in cfu/mL.

Tooth Element	T0	T1	T2
O 11 feldspathic- PROC.1CUP	1.5 × 10^8^	1.8 × 10^5^	5.2 × 10^5^
O 12 feldspathic- PROC.1CUP	1.5 × 10^8^	1.5 × 10^5^	6 × 10^5^
0 13 feldspathic- PROC. 2 AIR FLOW	1.5 × 10^8^	1.5 × 10^5^	6 × 10^5^
0 14 feldspathic- PROC. 2 AIR FLOW	1.5 × 10^8^	1.5 × 10^4^	4.5 × 10^5^
X 22 feldspathic- PROC.3 gum	1.5 × 10^8^	1.8 × 10^5^	5.4 × 10^5^
X 21 feldspathic- PROC.3 gum + H2o	1.5 × 10^8^	1.5 × 10^5^	4.5 × 10^5^
X 23 feldspathic- ctr.	1.5 × 10^8^	5 × 10^5^	1.5 × 10^6^
X 24 feldspathic- ctr.	1.5 × 10^8^	6 × 10^5^	1.8 × 10^6^

## Data Availability

Data sharing not applicable. No new data were created or analyzed in this study. Data sharing is not applicable to this article.
